# Microglial Hemoxygenase-1 Deletion Reduces Inflammation in the Retina of Old Mice with Tauopathy

**DOI:** 10.3390/antiox11112151

**Published:** 2022-10-30

**Authors:** José A. Fernández-Albarral, Elena Salobrar-García, José A. Matamoros, Cristina Fernández-Mendívil, Eric del Sastre, Lejing Chen, Rosa de Hoz, Inés López-Cuenca, Lidia Sánchez-Puebla, José M. Ramírez, Juan J. Salazar, Manuela G. Lopez, Ana I. Ramírez

**Affiliations:** 1 Instituto de Investigaciones Oftalmológicas Ramón Castroviejo, Grupo UCM 920105, IdISSC, Universidad Complutense de Madrid, 28040 Madrid, Spain; 2 Facultad de Óptica y Optometría, Departamento de Inmunología, Oftalmología y ORL, Universidad Complutense de Madrid, 28037 Madrid, Spain; 3 Instituto Teófilo Hernando for Drug Discovery, Department of Pharmacology, School of Medicine, Universidad Autónoma Madrid, 28029 Madrid, Spain; 4 Instituto de Investigación Sanitario (IIS-IP), Hospital Universitario de La Princesa, 28006 Madrid, Spain; 5 Facultad de Medicina, Departamento de Inmunología, Oftalmología y ORL, Universidad Complutense de Madrid, 28040 Madrid, Spain

**Keywords:** tauopathies, heme oxygenase 1 (HO-1), retina, microglia, macroglia, neuroinflammation, neurodegenerative diseases

## Abstract

Tauopathies such as Alzheimer’s disease are characterized by the accumulation of neurotoxic aggregates of tau protein. With aging and, especially, in Alzheimer’s patients, the inducible enzyme heme oxygenase 1 (HO-1) progressively increases in microglia, causing iron accumulation, neuroinflammation, and neurodegeneration. The retina is an organ that can be readily accessed and can reflect changes that occur in the brain. In this context, we evaluated how the lack of microglial HO-1, using mice that do not express HO-1 in microglia (HMO-KO), impacts retinal macro and microgliosis of aged subjects (18 months old mice) subjected to tauopathy by intrahippocampal delivery of AAV-hTau^P301L^ (TAU). Our results show that although tauopathy, measured as anti-TAUY9 and anti-AT8 positive immunostaining, was not observed in the retina of WT-TAU or HMO-KO+TAU mice, a morphometric study of retinal microglia and macroglia showed significant retinal changes in the TAU group compared to the WT group, such as: (i) increased number of activated microglia, (ii) retraction of microglial processes, (iii) increased number of CD68+ microglia, and (iv) increased retinal area occupied by GFAP (AROA) and C3 (AROC3). This retinal inflammatory profile was reduced in HMO-KO+TAU mice. Conclusion: Reduction of microglial HO-1 could be beneficial to prevent tauopathy-induced neuroinflammation.

## 1. Introduction

Neurodegenerative diseases (NDs) (Alzheimer’s disease (AD), Parkinson’s disease (PD), amyotrophic lateral sclerosis (ALS), among others) cause progressive degeneration of neurons leading to dementia. Their incidence increases with aging [1]. They also share some pathogenic mechanisms, such as DNA damage, epigenetic changes [2,3], metal accumulation, pathological protein aggregation and proteinopathy, oxidative stress, immune dysregulation, and microglial activation [4,5,6]. In NDs, the excessive accumulation of metals such as iron (Fe) can induce oxidative stress, mitochondrial dysfunction, DNA fragmentation, and apoptosis [2,7,8].

Tau protein accumulation (tauopathies) also occurs in many NDs, such as in AD and PD, among others [9,10]. Genetic mutations in tau protein can cause its hyperphosphorylation (p-tau), which alters microtubules and leads to the formation of pathological insoluble tau aggregates (neurofibrillary tangles), which are toxic to neurons [11,12]. These diseases are characterized by synaptic dysfunction, neuroinflammation, neuronal death, and dementia [13].

Neuroinflammation is a common process in NDs. In the inflammatory process of the central nervous system (CNS), glial cells (astroglia and microglia) play important roles and can respond and activate rapidly in the presence of any type of damage (reactive gliosis). However, chronic gliosis induces disease progression [14]. After injury, microglia proliferate, migrate to damaged areas, become more phagocytic, modify the expression of enzymes and receptors, and release a variety of inflammatory factors [15]. In addition, microglia activate and change their morphology (retract their processes, increase the soma size) and can adopt hyperamified, reactive, amoeboid, or even dystrophic forms. Dystrophic microglia is a phenotype especially related to aging and neurodegeneration and is associated with NDs [16,17,18,19]; it is characterized by cytoplasmic fragmentation and the release of proinflammatory cytokines associated with chronic neuroinflammation and parainflammation [20]. Microglia in the aging process and NDs show an increased inflammatory state and are referred to as primed microglia [21,22,23,24,25,26]. Activated microglia can exist in a continuum between two activation phenotypes, namely, the M1 and M2 [21,27]. The proinflammatory M1 state is associated with neurodegeneration and produces an intense inflammatory response characterized by the release of proinflammatory cytokines and inflammatory mediators, while in the M2 anti-inflammatory state, the release of anti-inflammatory cytokines and neurotrophic factors (BDNFs) occurs, contributing to neuronal survival [15,28,29,30]. Uncontrolled activation of the M1 phenotype can lead to a state of chronic inflammation that will cause progressive loss of neurons, as has been observed in some NDs [22,23].

After damage, astrocytes and Müller cells (macroglial cells in retina) respond by means of a process called reactive gliosis, which includes astrocyte proliferation and migration, an increase in soma size and processes, an increase in the number of astroglial processes [31], and an increase in the expression of gliofibrillary acidic protein (GFAP) [32]. This astrogliosis has been observed in NDs such as in EA [33] or PD [34]. Reactive astrocytes with a neurotoxic A1 phenotype have been described, which overexpress neuroinflammatory factors such as IL-1α and TNF-α, and complement proteins such as C1q or C3 [35], exhibiting neurotoxic and detrimental functions to the nervous system and destroying the synapse. These A1 astrocytes have been detected in several ND models, such as ALS, AD, prion disease, or Huntington’s disease [36].

The retina, as part of the CNS, is considered a window to the brain [37]. It has been observed that several NDs affecting the brain have retinal involvement and even precede brain damage [37]. Therefore, the study of the retina may be useful to indicate the progression of these NDs [38,39,40,41].

Within NDs, tauopathies can have retinal involvement. In the retina, tau protein is expressed in the plexiform layers [42]. In animal models of AD, increased tau phosphorylation has been observed in these layers compared to normal retinal tissues, in addition to microglial activation, loss of retinal ganglion cells (RGCs), and impaired retinal function. In some studies, these changes have preceded the formation of tau aggregates in the brain [43,44].

As we mentioned above, neuroinflammation is a key process in NDs, and therefore strategies for the control of inflammation are being developed. Iron and the neuroinflammatory process are bidirectionally related in such a way that iron modifies the inflammatory phenotype of microglia and, in turn, microglia secrete diffusible mediators that cause remodeling of neuronal iron homeostasis and also regulate the entry of iron into the brain [45]. Iron homeostasis involves a molecule called heme oxygenase (HO). This enzyme participates in the catabolism of the heme group, giving rise to three products, namely, carbon monoxide (CO); biliverdin, which is rapidly converted to bilirubin; and labile iron (Fe^2+^) [46,47]. HO-1 is an inducible enzyme known for its antioxidant, anti-inflammatory, and neuroprotective effects [48,49,50]. Under physiological conditions, HO-1 expression is found at very low levels in the CNS, but this expression can be rapidly induced, preferentially in glial cells, in response to stress and various oxidative stimuli, including metals, light exposure, and inflammatory cytokines (TNF-α, β-amyloid) [51,52,53]. However, chronic systemic inflammation that occurs with aging and in NDs increases HO-1 expression, mainly mediated by activated microglia [54,55], and has been related to disease progression [56]. This is associated with an accumulation of iron derived from HO-1 catabolism, together with alterations in iron transport and metabolism, contributing to exacerbate the neurodegenerative effect [52,57,58].

Genetic deletion of HO-1 or pharmacological inhibition of HO-1 activity may constitute interesting therapeutic approaches to reduce age-related pathological features and delay the development of NDs [55].

Knowing that the retina reflects the pathology of the brain and that microglial HO-1 deletion was protective in old subjects subjected to an inflammatory stimuli, the aim of this study was to evaluate how microglial deletion of HO-1 could impact the inflammatory process in the retina (analyzing microglia, astrocytes, and Müller glia) of old mice with brain tauopathy.

## 2. Materials and Methods

### 2.1. Animals and Experimental Groups

All animals were housed in the animal facility of the Faculty of Medicine of the Autónoma University of Madrid, subjected to a 12 h light/12 h dark cycle. The animals had access to food and water ad libitum. Every effort was made to minimize animal suffering and reduce the number of animals used. All experimental procedures were performed following the Guide for the Care and Use of Laboratory Animals and were previously approved by the Institutional Ethics Committee of the Autonomous University of Madrid and the Autonomous Community of Madrid, Spain (PROEX 218.5/20) following the European Guidelines for the use and care of animals of animals for research in accordance with the European Union Directive of 22 September 2010 (2010/63/EU) and with the Spanish Royal Decree of 1 February 2013 (53/2013).

Aged 15-month-old male C57BL/6 mice were used and distributed into the following study groups: Wild type (WT) control group (*n* = 6); Tauopathy (TAU) group (*n* = 6); HO-1 knockout group (HMO-KO) (*n* = 6); HO-1 knockout group with tauopathy (HMO-KO+TAU) (*n* = 6).

### 2.2. Generation of Hemoxygenase-1 Knockout Animals for the HMO-KO and HMO-KO+TAU Groups

Microglial cell-specific knockout (KO) mice for the HMOX1 gene (LyzMcreHMOX1f/f: HMOX1M-KO) were generated using the cre/LoxP system at the Autónoma University of Madrid. For this purpose, LyzMcre mice (The Jackson Laboratory: B6.129P2-Lyz2tm1(cre)Ifo/J, stock: 004781), which carry the cre recombinase under the control of a myeloid promoter; and HMOX1LoxP mice (RIKEN BioResource Center, stock.: RBRC03163) were crossed, giving rise to heterozygous offspring for both the cre recombinase and the LoxP site. Heterozygous mice were crossed and offspring homozygous for the LoxP site carrying the cre recombinase were used for experiments (HMO-KO). In these mice, cre recombinase mediates a specific deletion of the HMOX1 gene flanked by LoxP recombination sites in myeloid cells and thus in brain microglial cells. The WT group animals presented the LoxP recombination sites but not the Cre recombinase; therefore, in these mice, the deletion of the gene of interest does not occur, being considered WT control mice or littermates.

### 2.3. Generation of Animals with Tauopathy: TAU and HMO-KO+TAU Groups

Mice belonging to the TAU Group and HMO-KO+TAU Group were anesthetized with isofluorane (ISOFLO Isoflurane 100% *w*/*w*, Zoetis SL, Alcobendas, Madrid, Spain) at 5% under oxygen. Mice were placed in a stereotaxic and for intrahippocampal injections, a cranial midline incision was made, and the skull was drilled 1.94 mm posterior and 1.4 mm lateral to the bregma on both sides with a micromanipulator and a microdrill. For the tauopathy model, adenoassociated particles with the human tau protein mutated at P301L under the neuronal specific promoter, synapsin I (AAV-hTau (2 × 1012 VP/mL)), were injected into both hippocampi at 1.8 mm below the dura mater (0.1 μL/min) with a Hamilton automatic syringe. Syringes were held in position for 2 min after each injection. For WT and HMO-KO mice, adeno-associated particles lacking the mutated human tau protein and containing the eGFP (enhanced green fluorescent protein) snitch gene under the SYN1 promoter (AAV-eGFP (2 × 1012 VP/mL) were injected.

### 2.4. Immunohistochemistry

Mice were deeply anesthetized with 5% isofluorane. They were then transcardially perfused through the ascending aorta, first with saline (PBS) and then with 4% paraformaldehyde (PFA) in 0.1 M phosphate buffer (PBS, pH 7.4).

Subsequently, eyeballs were removed. The orientation of each eye was carefully maintained with a suture given in the upper eyelid before enucleation of the eyeballs. During eye dissection, the insertion of the rectus muscle and the nasal caruncle were used as additional landmarks. The eyes were postfixed for 24 h in the same fixative and then transferred to a 0.1 M PBS solution at 4 °C. The retinas were then separated from the rest of the ocular layers, and wholemounts were made. Subsequently, the retinas were cryoprotected in sucrose at increasing concentrations (10%, 20%, and 30%) at 1 h, 2 h, and overnight, respectively. They were then frozen in liquid nitrogen and kept at −80 °C until use.

In addition to the eyeballs, brains were also removed and kept overnight at 4 °C in 4% PFA. Then, the tissue was cryoprotected for 2 days in 30% sucrose, and 40 μm thick coronal slices were cut using a sliding microtome and collected in phosphate buffer (PB) 0.1 M.

For the immunohistochemical labeling of retinal wholemounts, retinas from left eyes were used for the microglial study, and retinas from right eyes were used for the macroglial study. Triple immunofluorescences were performed. In the right eyes the following primary antibodies were used: anti-GFAP to label macroglial cells (astrocytes and Müller glia), anti-C3 to label A1 phenotype astrocytes, and anti-TAUY9 to label phosphorylated tau protein from human tau protein (Table 1). In the left eyes, the antibodies used were: anti-Iba-1 (a microglial marker), anti-CD68 to label activated microglia with phagocytic capacity, and anti-AT8 to label injected human phosphorylated tau protein and mouse tau protein. Secondary antibodies that bound to their corresponding primary antibodies were used. Each secondary antibody was conjugated with a determined fluorochrome, as indicated in Table 1, which allowed for their detection during a double-labelling fluorescent immunohistochemistry study.

In addition, three negative controls were made to ensure the specificity of immunolabeling. In the first one, the primary antibodies were not added, and the retinas were incubated only in the secondary antibodies with their respective diluents. In the second control, the retinas were incubated in the primary antibodies with their diluents. In the third control, only the primary and secondary antibody diluents were added to the retinas to analyze the endogenous fluorescence of the tissue.

For immunofluorescence assays of brain sections, these were blocked with 5% goat or donkey-serum for 2 h. Afterwards, sections were incubated at 4 °C with the primary antibodies (Ty9: 1:1000 and AT8: 1:500) (Table 1). Sections were washed three times and then incubated with the appropriate secondary fluorescent antibodies for 1 h (Table 1). After three washes (Hoechst was added in the second wash: 1 μL/mL), sections were mounted and images taken using a Zeiss Axio Imager M.2 fluorescence optical microscope (Carl Zeiss AG, Oberkochen, Germany).

### 2.5. Morphometric Analysis of Retinal Wholemounts

Retinal wholemounts were analyzed and photographed using a Zeiss Axio Imager M.2 fluorescence optical microscope (Carl Zeiss AG, Oberkochen, Germany) equipped with the appropriate filters for different emission spectra: Alexa Fluor 488 (filter set 10, Zeiss), Alexa Fluor 594 (filter set 64, Zeiss), and Alexa Fluor 405 (filter set 49, Zeiss). The microscope was associated with the Apotome-2 module (Carl Zeiss AG, Oberkochen, Germany). Microphotographs were taken using a high-resolution Axio Cam 503 Mono digital camera (Carl Zeiss AG, Oberkochen, Germany) attached to the microscope. The Apotome-2 module allows high-quality photographs to be taken of thick tissue samples. Imaging of thick samples in fluorescence microscopy is compromised by signals originating outside the focal plane, causing a reduction in contrast and resolution of the axial dimension (*Z*-axis). The Apotome allows imaging like an optical section, improving contrast and resolution. Its principle is based on the theory of interferometry, projecting a grating on the focal plane of the objective that moves to three different positions on the sample. These images are then processed in real time by the software ZEN2 (Carl Zeiss AG, Oberkochen, Germany) of the microscope, and everything below and above the focus range is eliminated by the software. The result is an optimized image that resembles an optical section of the sample in the plane of focus.

#### 2.5.1. Tau Protein Expression

In the retinas in which human tau had been injected and after immunohistochemical labelling with TAUY9 and AT8 antibodies, all retinal wholemounts were systematically analyzed (superior, inferior nasal, and temporal), and microphotographs were taken at 20×.

#### 2.5.2. Microglial Characterization

For the qualitative and quantitative study of microglia, retinal wholemounts labelled with the primary anti-Iba-1 and anti-CD68 antibodies were used.

Microglial cells are arranged in the retina forming several plexuses: in the photoreceptor outer segment layer (OS), outer plexiform layer (OPL), inner plexiform layer (IPL), and nerve fiber layer (NFL)–ganglion cell layer (GCL). The microglia in the latter two layers are near each other and difficult to distinguish, so the layers were analyzed together and referred to as the inner retinal layer complex (ILC). Therefore, for quantitative analysis of Iba-1+ cells, three different plexuses were analyzed in the *Z*-axis of the retina: OS, OPL, and ILC.

Twelve areas were photographed at 20x in each plexus, 3 for each of the four sectors (superior, inferior, nasal, and temporal). Since 3 plexuses (OS, OPL, and ILC) were analyzed, a total of 12 × 3 = 36 microphotographs was obtained for each retinal wholemount. As 6 retinas per experimental group were analyzed for cellular measurements, a total of (36 × 6) 216 microphotographs was obtained and analyzed for each experimental group. The photographs were taken at 20× magnification, providing an area of 0.1502 mm^2^ per field. For the *Z*-axis images, the Extended Focus module of the ZEN2 software (Carl Zeiss AG, Oberkochen, Germany) was used, which allowed the extraction of focus details from images at different focal planes and the generation of an image with a greater depth of focus. Images were taken first in the red channel (Iba-1+), then in the green channel (CD68+), and the mixed image of both channels was obtained to obtain the colocalization of both antibodies.

##### Number of Microglia Iba-1+

On the images previously obtained at 20×, Iba-1+ microglia somas in each microphotograph were manually counted using the interactive manual counting tool included in the ZEN2 software, which is incorporated in the microscope.

In addition, in the OS layer, the number of dystrophic microglia (cells with fragmented cytoplasm) and the number of activated microglia (thickened or amoeboid), morphotypes that were only observed in this retinal layer, were also counted manually in the same images.

##### Number of Microglia Iba-1+/CD68+

The number of microglia with Iba-1+/CD68+ phagocytic capacity (colocalization of red and green channels) was counted manually in each microphotograph, using the interactive manual counting tool included in the ZEN2 software (Carl Zeiss AG, Oberkochen, Germany), which is incorporated in the microscope. The counting of dystrophic Iba-1+ microglia with CD68+ colocalization was also done in the OS layer.

Since the microglial cells had different levels of CD68+ labelling, a grading of phagocytic capacity was incorporated: (i) Low phagocytic capacity, where microglia had only a small, almost inappreciable point of CD68+ labelling. This grade was not considered in the counting, as it was present in most of the microglial cells. (ii) Medium phagocytic capacity (M). The microglia have one large spot or two or more spots of CD68+ labelling. (iii) High phagocytic capacity (H). Microglia have CD68+ labelling in almost the whole cell.

##### Area of Soma and Arbor Area of Iba-1+ Cell

The measurement of the soma area was performed on the OPL and ILC images using a semiautomatic method with the Interactive Measurement tool of the ZEN2 software (Carl Zeiss AG, Oberkochen, Germany). This tool allows thorough manual delimitation of the soma contour to know the value in μm^2^. For the measurement of the soma area, 3 to 5 microglia were selected in each image at 20×.

Measurements of the arbor area were performed with a procedure like that of the soma area, but we delimited the cell outline by joining the most distal tips of the primary and secondary processes. For arbor area measurements, all microglia that appeared complete in each image were analyzed at 20×.

These measurements were not performed in the OS because in this layer microglia possess great morphological variability, not possessing the classical branched appearance as in the OPL and ILC.

#### 2.5.3. Macroglial Characterization

Retinal wholemounts labelled with the primary anti-GFAP and anti-C3 antibodies were used for the qualitative and quantitative study of macroglia. Twelve areas were systematically selected from each retinal whole mount, 3 for each sector (superior, inferior, nasal, and temporal), and photographed under the same capture parameters.

##### Retinal Area Occupied by GFAP+ Immunolabeling (AROA)

The AROA was quantified at 20× magnification in the nerve fiber layer–ganglion cell layer (NFL–GCL) where astrocytes are located. For this, a semi-automatic measurement was performed with the help of an algorithm developed in MATLAB^®^, which had a tool called “threshold“; a variable thresholding level based on a grey scale was applied to each image. The algorithm returned a value representing the percentage of the AROA with respect to the total area of the image. Each image captured at 20× corresponded to an area of 0.1502 mm^2^.

##### Retinal Area Occupied by C3+ Immunolabeling (AROC3)

For the study of AROC3, images were taken at 40× in the nerve fiber layer–ganglion cell layer (NFL–GCL), where astrocytes are located, of 3 different areas of each sector (superior, inferior, nasal, and temporal), and the same procedure used to measure AROA was employed. This was done at this magnification to prevent any macrophages, which are also labelled with C3, from being in the measurement field and falsifying the count. Each image captured at 40× corresponded to an area of 0.0376 mm^2^.

### 2.6. Statistical Analysis

The assessment of normal distribution of data was carried out with the Shapiro–Wilk test. Levene’s test was used to assess the homogeneity of variance between groups. Statistical significance between groups was assessed depending on the distribution by one- or two-way ANOVA with the Tukey post hoc test, for multiple comparisons, or the Kruskal–Wallis test. ANOVA post hoc tests were carried out only if F had a *p* < 0.05, and no significant variance inhomogeneity was found within analyzed groups. Differences were considered statistically significant for *p* values < 0.05. Statistical analysis was carried out with GraphPad Prism v.9 (GraphPad Software, La Jolla, CA, USA), and the same software was used to design graphs. The notations used for the different levels of significance were: * *p* < 0.05, ** *p* < 0.01, *** *p* < 0.001, **** *p* < 0.0001.

## 3. Results

### 3.1. Tau Expression in Retinal Tissue

**Figure 1:**
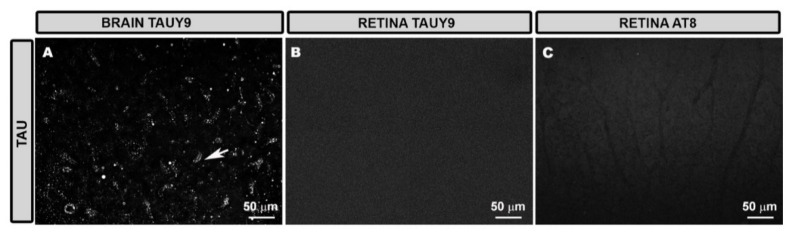
TAUY9 labeling in the hippocampus (arrow) (**A**), absence of TAUY9 (**B**), and AT8 (**C**) labelling in retinal tissue. (**B**,**C**) correspond to the inner complex retinal layers (ICL) of animals from the TAU group.

After injecting the adenoassociated virus containing the human tau protein with the P301L mutation (AAV-hTau) in the hippocampus (**TAU group**), TAUY9+ immunohistochemical labelling, which identifies human phosphorylated tau protein, was observed in the cells of this tissue (Figure 1A). However, when analyzing retinas, TAUY9 staining was not found in any of the TAU groups analyzed (Figure 1B) nor when using AT8 antibody labelling, which measures human and mouse phosphorylated tau protein (Figure 1C).

### 3.2. Morphology and Distribution of Retinal Microglia in the Different Study Groups

#### 3.2.1. Outer Segment Layer (OS)

**Figure 2:**
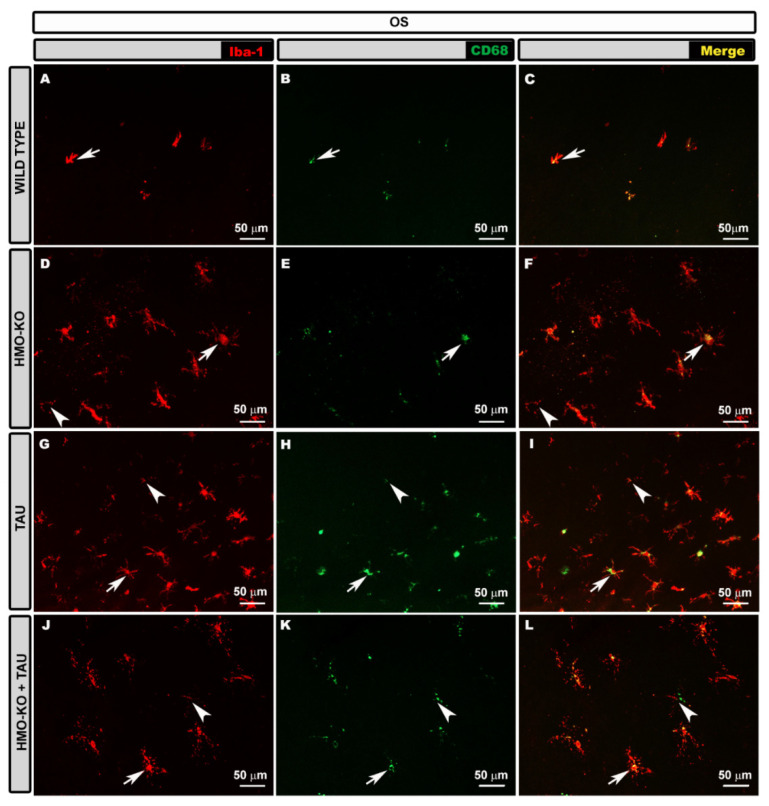
Retinal wholemount. Double immunolabeling with anti-Iba-1 (red) and anti-CD68 (green) in OS layer in the different study groups: WT (**A**–**C**), HMO-KO (**D**–**F**), TAU (**G**–**I**), HMO-KO+TAU (**J**–**L**); 20× magnification. Microglia with CD68+ labelling (arrows). Dystrophic microglia (arrowheads). The images correspond to the retinal periphery. Number of retinas used in the experiment: WT *n* = 6, HMO-KO *n* = 6, TAU *n* = 6, and HMO-KO+TAU *n* = 6.

In the OS layer, in the **WT group**, microglia were not evenly distributed in the retina, being preferentially located in the peripheral areas. Iba-1+ cells with a different morphology were observed. There were cells with an ovoid soma from which processes originated from the same point, and they were arranged perpendicular to the retinal surface; and other cells were elongated, with a bipolar appearance, parallel to the retinal surface. We found other cells of similar appearance, but with thickened somas and more retracted processes (Figure 2A–C). We also observed cells in which the cytoplasmic processes were fragmented, and we frequently observed in them structures of beads or spheroids; these cells corresponded to dystrophic microglia.

In the **HMO-KO group**, more microglia were observed than in the WT, and the cells had a similar morphological appearance, but with thicker cell bodies and more branched and thickened processes. Numerous dystrophic microglia were also observed (Figure 2D–F). All these cells were preferentially located in the peripheral areas of the retina.

In the **TAU group**, more cells were apparently observed than in the WT group and in the HMO-KO group (Figure 2G–I). The morphology was similar to that of the HMO-KO group; however, the somas were thicker, and the processes were more branched, although more retracted. In general, many more dystrophic microglia were observed than in the rest of the groups, especially with respect to the WT group and the HMO-KO group.

In the **HMO-KO+TAU group**, the microglia were more similar to those in the HMO-KO group (Figure 2J–L), the somas were thickened, and the processes were branched, elongated, and less retracted than in the TAU group. In addition, fewer microglia were apparently observed than in the TAU group. Although dystrophic microglia were present, there appeared to be fewer of them than in the TAU group.

#### 3.2.2. Outer Plexiform Layer (OPL)

**Figure 3:**
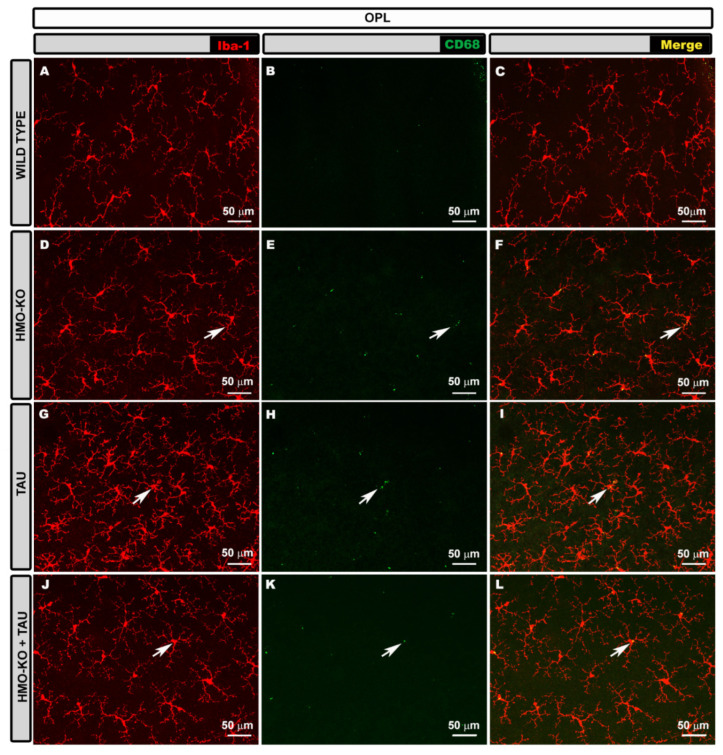
Retinal wholemount. Double immunostaining with anti-Iba-1 (red) and anti-CD68 (green) in OPL in the different study groups: WT (**A**–**C**), HMO-KO (**D**–**F**), TAU (**G**–**I**), HMO-KO+TAU (**J**–**L**); 20× magnification. Arrows indicate microglial cells with CD68+ labelling. Number of retinas used in the experiment: WT *n* = 6, HMO-KO *n* = 6, TAU *n* = 6, and HMO-KO+TAU *n* = 6.

In the OPL, the microglia of the **WT group** formed a regular mosaic-like plexus. The cells had small somas from which elongated primary processes emerged, which in turn divided into secondary and tertiary processes, giving the cell a branched appearance (Figure 3A–C).

In the **HMO-KO group**, the microglia formed a plexus similar to the WT group, but the cells had thicker somas and processes (Figure 3D–F).

In the **TAU group**, the microglial plexus was denser because the microglial cells were closer together, and there appeared to be more microglial cells. In addition, the somas appeared more thickened, as in the HMO-KO group, but the processes were more retracted than in the WT group and the HMO-KO group (Figure 3G–I).

In the **HMO-KO+TAU group**, the microglial plexus was more similar to that of WT and HMO KO, the microglia were less proximal, and their processes were less retracted than in the TAU group. However, the somas and processes were thickened, as in the HMO-KO group (Figure 3J–L).

#### 3.2.3. Inner Complex Layers (ICL)

**Figure 4:**
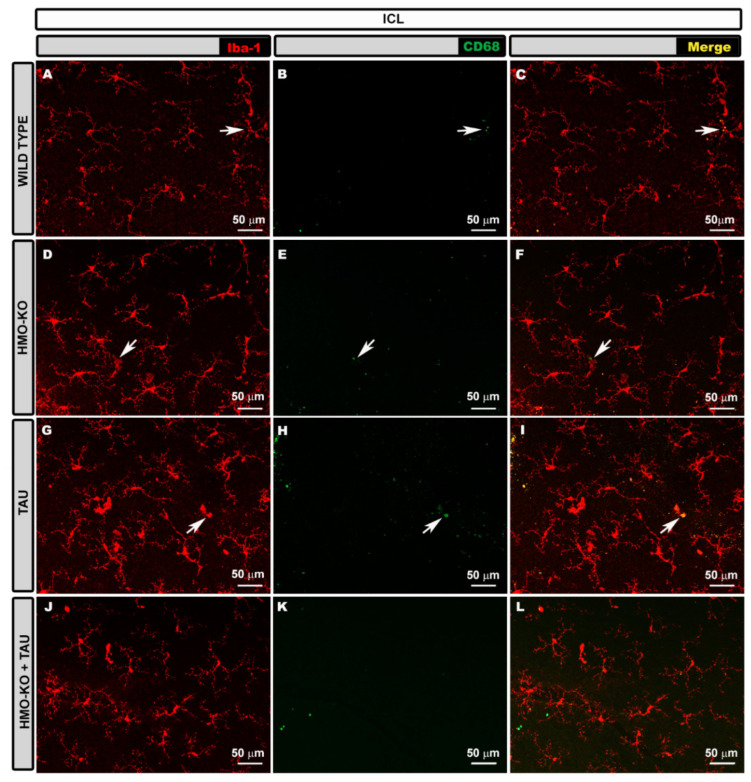
Retinal wholemount. Double immunostaining with anti-Iba-1 (red) and anti-CD68 (green) in ICL in the different study groups: WT (**A**–**C**), HMO-KO (**D**–**F**), TAU (**G**–**I**), HMO-KO+TAU (**J**–**L**); 20× magnification. Arrows indicate microglial cells with CD68+ labelling. Number of retinas used in the experiment: WT *n* = 6, HMO-KO *n* = 6, TAU *n* = 6, and HMO-KO+TAU *n* = 6.

In the ICL, in the **WT group**, the microglial plexus had a less regular appearance than in the OPL. The microglia also possessed small somas, from which primary, secondary, and tertiary processes emerged (Figure 4A–C).

In the **HMO-KO group**, the microglial plexus was similar to that of the WT group, but the microglial cells had thicker somas and processes (Figure 4D–F).

In the **TAU group**, the plexus was similar to that of the previous groups, but many microglia showed retraction of their processes, and even amoeboid cells were observed (Figure 4G–I).

In the **HMO-KO+TAU group**, the plexus was similar to the rest of the groups, but the appearance of the somas and processes were more similar to the WT, as they were less thickened than in the TAU group. In addition, the processes showed less retraction than in the TAU group (Figure 4J–L).

#### 3.2.4. Microglial CD68 Expression

In the **OS layer**, in the WT group, some cells with some CD68+ vesicles were observed (Figure 2B,C). In the HMO-KO group, these cells with CD68+ vesicles were increased with respect to the WT group (Figure 2E,F). The group in which more CD68+ labelling was observed was the TAU group, where many cells had CD68+ labelling occupying the entire soma and processes (Figure 2H,I). This immunolabeling decreased in the HMO-KO+TAU group with respect to the TAU group, as the labelling was more punctate and did not fill the entire cell (Figure 2K,L).

In **OPL**, labelling was observed as small CD68+ vesicles within the cell. In the WT group, labelling was scarce (Figure 3B,C). This labelling increased in the rest of the groups, with more CD68+ vesicles being observed in the HMO-KO and TAU groups (Figure 3E,F). In the latter, some thicker vesicles were also observed (Figure 3H,I). In the HMO-KO+TAU group, smaller and sparser vesicles were observed than in the TAU group (Figure 3K,L).

In the **ICL**, labelling was scarce in the WT group (Figure 4B,C) and increased in the HMO-KO group (Figure 4E,F), but CD68+ vesicles were more abundant and thicker in the TAU group (Figure 4H,I), although they decreased considerably in the HMO-KO+TAU group (Figure 4K,L).

#### 3.2.5. Quantitative Study of Retinal Microglial Cells

##### Microglial Cell Numbers

**Figure 5:**
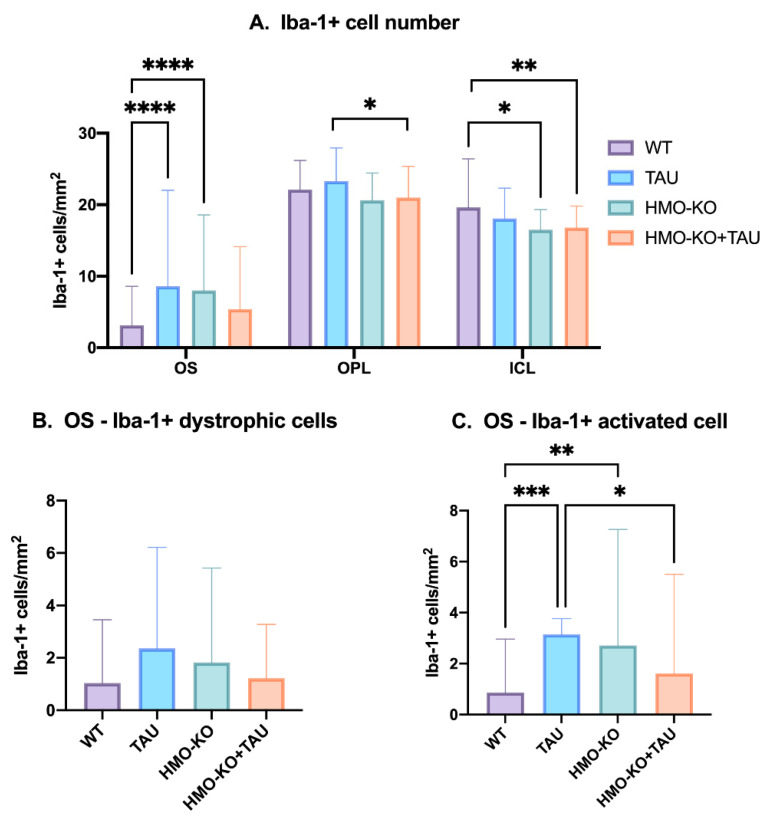
Microglial cell numbers. (**A**) Number of Iba-1+ microglial cells per retinal area of 0.1502 mm^2^ in the different retinal layers in each of the study groups; two-way ANOVA. (**B**) Number of dystrophic Iba-1+ cells in OS layer in the different study groups; one-way ANOVA. (**C**) Number of activated Iba-1+ cells in OS layer in the different study groups; one-way ANOVA. All data are expressed as mean value (±SD). * *p* < 0.05, ** *p* < 0.01, *** *p* < 0.001, **** *p* < 0.0001. Number of retinas used in the experiment: WT *n* = 6, HMO-KO *n* = 6, TAU *n* = 6, and HMO-KO+TAU *n* = 6.

When analyzing the **number of microglial cells** per retinal layer (Figure 5A), in the **OS layer** in both the TAU and HMO-KO groups there was a significant increase in the number of Iba-1+ cells with respect to the WT group (*p* < 0.0001); this was not found in the HMO-KO+TAU group. In the **OPL,** there was a significant decrease in the number of Iba-1+ cells in the HMO-KO+TAU group with respect to the TAU group (*p* < 0.05). In **ICL,** there was a significant decrease of Iba-1+ cells in the HMO-KO group (*p* < 0.05) and the HMO-KO+TAU group (*p* < 0.01) with respect to WT.

##### Number of Dystrophic Iba-1+ Microglia and Iba-1+ Microglia Activated in OS Layer

In the **OS layer,** there was a non-significant increase of dystrophic microglia in the TAU group with respect to the WT group (*p* > 0.05). Additionally, a non-significant decrease was found in the HMO-KO+TAU group with respect to the TAU group (Figure 5B).

In the number of activated Iba1+ cells in the **OS layer**, there was a significant increase in activated microglia in the TAU group (*p* < 0,001) and in the HMO-KO (*p* < 0.01) with respect to the WT group, and a significant decrease in the HMO-KO+TAU group with respect to the TAU group (*p* < 0.05) (Figure 5C).

##### Microglial Arbor Area

**Figure 6:**
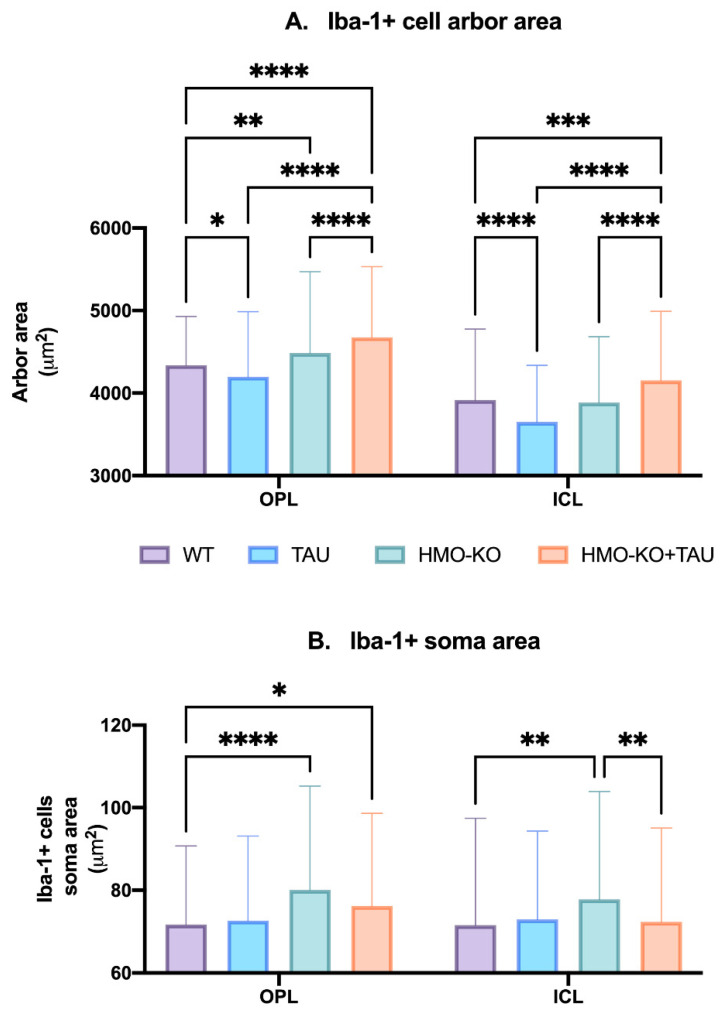
(**A**) Arbor area of Iba-1+ microglia in OPL and ICL in the different study groups. (**B**) Area of the Iba-1+ soma in OPL and ICL in the different study groups. Two-way ANOVA. Data expressed as mean value (±SD). * *p* < 0.05, ** *p* < 0.01, *** *p* < 0.001, **** *p* < 0.0001. Number of retinas used in the experiment: WT *n* = 6, HMO-KO *n* = 6, TAU *n* = 6, and HMO-KO+TAU *n* = 6.

The analysis of the **OPL** and **ICL** (Figure 6A) showed a significant decrease in the area of microglia arborization (indicating process retraction) in the TAU group compared to the WT group (*p* < 0.05). However, in the HMO-KO+TAU group, the area of arborization was significantly greater than in the WT group (*p* < 0.0001 in OPL and *p* < 0.001 in ICL), in the TAU group (*p* < 0.0001 in both layers), and in the HMO-KO group (*p* < 0.001 in both layers).

##### Microglial Soma Area

In **OPL**, there was a significant increase in the Iba-1+ soma area in the HMO-KO group and the HMO-KO+TAU group with respect to the WT group (*p* < 0.0001 and *p* < 0.05, respectively) (Figure 6B).

In **ICL**, there was a significant increase in the soma area of Iba-1+ cells in the HMO-KO group with respect to the WT group *p* < 0.01). There was a significant decrease in the soma area in the HMO-KO+TAU group with respect to the HMO-KO group (*p* < 0.01). No significant differences were observed between the TAU group and the HMO-KO+TAU group with the WT group (Figure 6B).

##### Number of Iba-1+/CD68+ Cells

**Figure 7:**
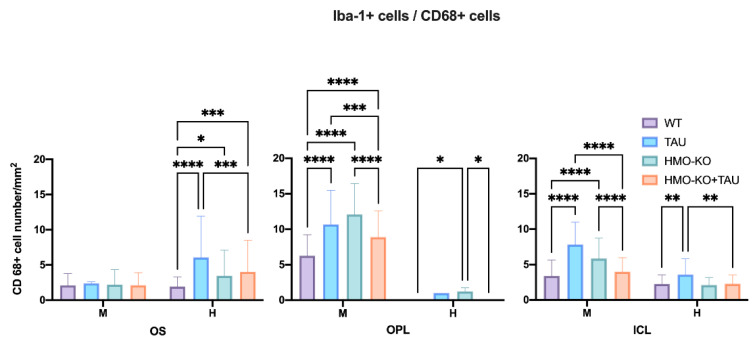
Number of Iba-1+/CD68+ microglial cells per retinal area of 0.1502 mm^2^ in the different retinal layers in each of the study groups, expressed as mean value (±DS). Two-way ANOVA; H: high phagocytic capacity; M: mean phagocytic capacity; * *p* < 0.05, ** *p* < 0.01, *** *p* < 0.001, **** *p* < 0.0001. Number of retinas used in the experiment: WT *n* = 6, HMO-KO *n* = 6, TAU *n* = 6, and HMO-KO+TAU *n* = 6.

In the **OS layer**, there was a significant increase in the number of CD68+ cells with high phagocytic capacity (H) in the TAU group with respect to the WT group (*p* < 0.0001) and HMO-KO+TAU (*p* < 0.001). Compared with the WT group, there was also a significant increase in the number of CD68+ cells with high phagocytic capacity in the HMO-KO (*p* < 0.05) and in the HMO-KO+TAU groups (*p* < 0.001) (Figure 7).

In **OPL**, there was a significant increase in the number of CD68+ cells with mean (M) phagocytic capacity in all three groups (TAU, HMO-KO, HMO-KO+TAU) with respect to the WT group (*p* < 0.0001). There was a significant decrease in the number of CD68+ cells with mean phagocytic capacity (M) in the HMO-KO+TAU group with respect to the TAU group (*p* < 0.001) and the HMO-KO group (*p* < 0.0001) (Figure 7). With respect to the number of CD68+ cells with high phagocytic capacity (H), there was a significant increase in the HMO-KO group with respect to the WT group and the HMO-KO+TAU group (*p* < 0.05, in both cases).

In **ICL**, there was a significant increase in the number of CD68+ cells with mean phagocytic capacity (M) in the TAU and HMO-KO groups with respect to the WT group (*p* < 0.0001). There was a significant decrease in the number of CD68+ cells with mean phagocytic capacity (M) in the HMO-KO+TAU group with respect to the TAU group (*p* < 0.0001) and the HMO-KO group (*p* < 0.0001). In the number of CD68+ cells with high phagocytic (H) capacity, there was also a significant increase in the TAU group with respect to the WT group (*p* < 0.01), and in the HMO-KO+TAU group with respect to the TAU group (*p* < 0.01) (Figure 7).

**Figure 8:**
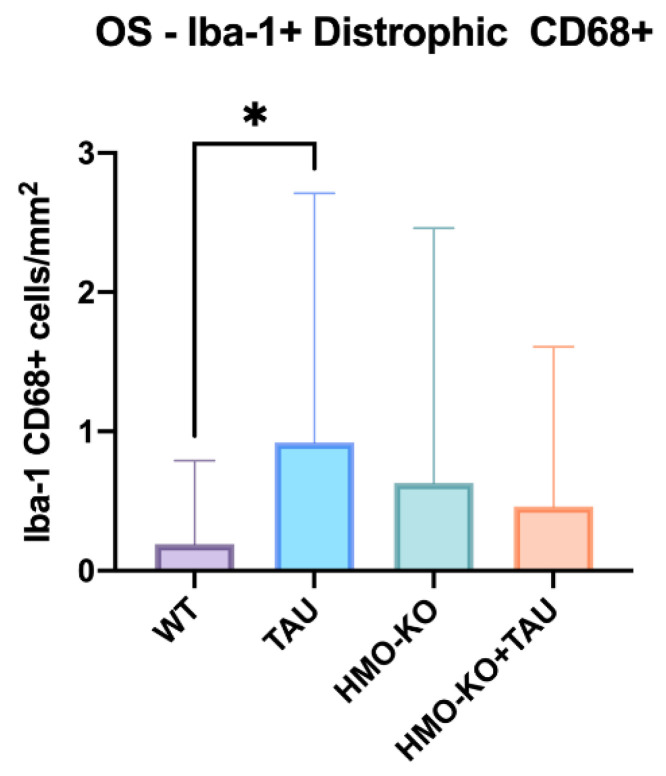
Number of Iba-1+/CD68+ dystrophic microglial cells per retinal area of 0.1502 mm^2^ in the OS layer in each of the study groups, expressed as mean value (± SD). One-way ANOVA; * *p* < 0.05. Number of retinas used in the experiment: WT *n* = 6, HMO-KO *n* = 6, TAU *n* = 6, and HMO-KO+TAU *n* = 6.

In the **OS layer** there was a significant increase of the **CD68+ dystrophic cells** in the TAU group compared with the WT group (*p* < 0.05), the increase being not significant compared to the other two groups (Figure 8).

### 3.3. Morphology and Distribution of Retinal Macroglia in the Different Study Groups

#### 3.3.1. Morphological Study Using GFAP Antibody

**Figure 9:**
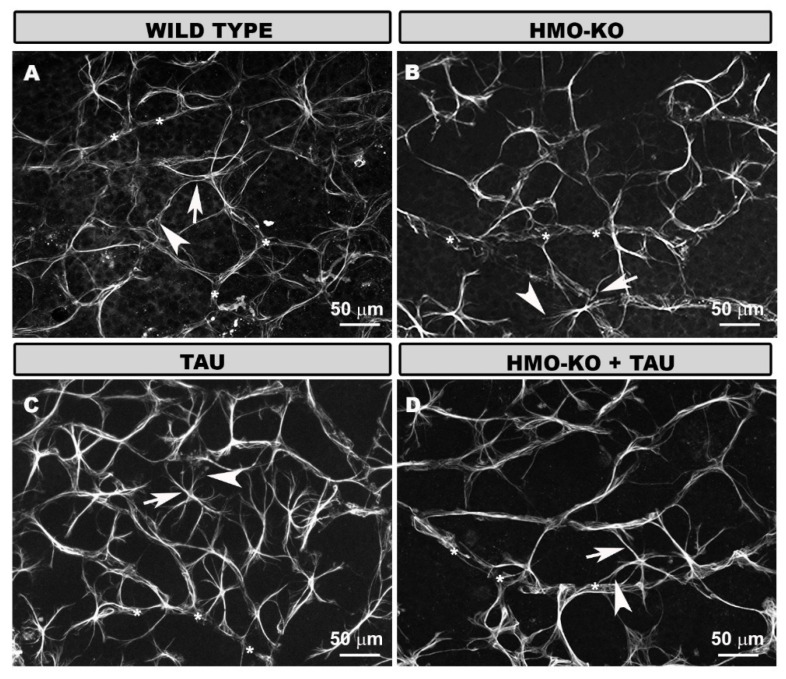
Retinal wholemount. GFAP+ astrocytes in the different study groups: WT (**A**), HMO-KO (**B**), TAU (**C**), HMO-KO+TAU (**D**); 20× magnification. Arrows point to primary astrocyte processes; arrowheads point to secondary astrocyte processes. Asterisks (*) demarcate the course of blood vessels. Number of retinas used in the experiment: WT *n* = 6, HMO-KO *n* = 6, TAU *n* = 6, and HMO-KO+TAU *n* = 6.

In the 15-month-old **WT group**, astrocytes formed a honeycomb-like plexus of stellate-shaped cells distributed from the optic disc to the periphery of the retina in the NFL–GCL. In this plexus, the astrocytes could distinguish themselves from each other and send processes toward the blood vessels or arrange their somas on the blood vessels following the vascular trajectory (Figure 9A).

**Figure 10:**
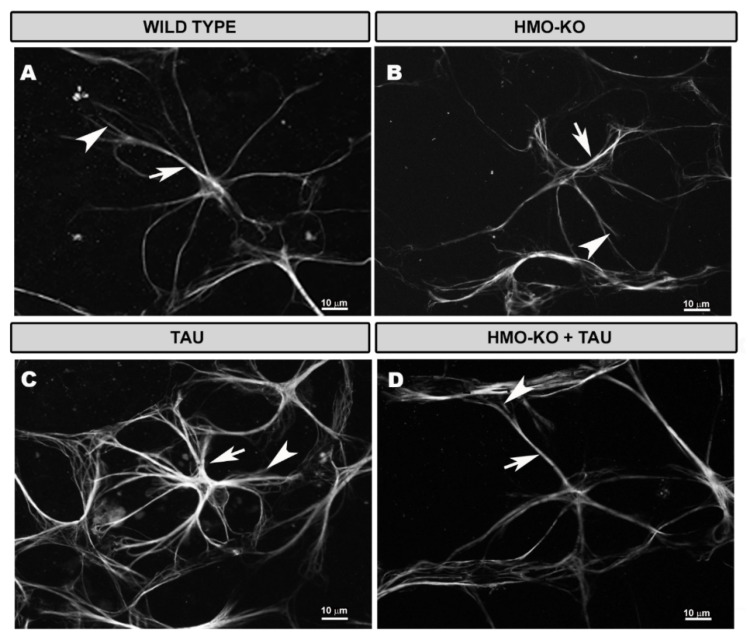
Retinal wholemount. GFAP+ astrocytes in the different study groups: WT (**A**), HMO-KO (**B**), TAU (**C**), HMO-KO+TAU (**D**); 63× magnification. Arrows indicate primary astrocyte processes; arrowheads indicate secondary astrocyte processes. Number of retinas used in the experiment: WT *n* = 6, HMO-KO *n* = 6, TAU *n* = 6, and HMO-KO+TAU *n* = 6.

At a higher magnification, the astrocytes had a rounded cell body from which primary processes branched into smaller secondary processes, which could join other astrocyte processes or the surface of blood vessels (Figure 10A).

In the **HMO-KO group**, the astrocytes had characteristics similar to those of the WT group (Figure 10B), and the plexus they formed resembled that observed in WT group animals (Figure 9B).

In the **TAU group**, astrocytes were observed to have thicker somas and primary processes than in the WT group; in addition, they had numerous secondary processes, giving the cell a more branched appearance, and occupying more space in the retina (Figure 10C); this resulted in a denser astroglial plexus and closer honeycomb cells (Figure 9C).

In the **HMO-KO+TAU group,** the astrocytes were thinner and had fewer secondary processes compared to the TAU group (Figure 10D). Both the astrocytes and the astroglial plexus (which was less dense) were more similar to that of the WT group (Figure 9D).

#### 3.3.2. Morphological Study Using C3 Antibody

**Figure 11:**
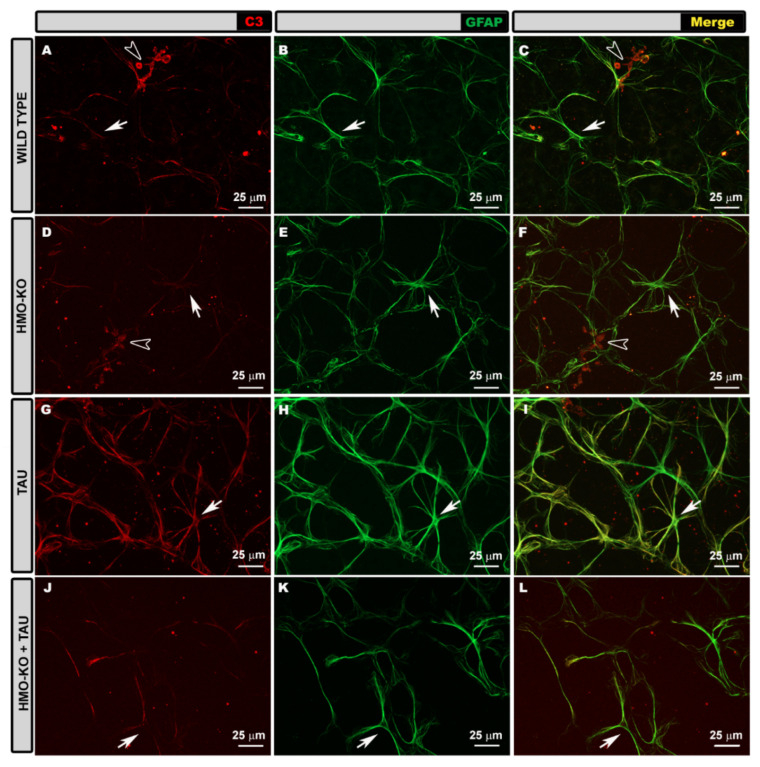
Retinal wholemount. Double immunohistochemical staining with GFAP (green) and C3 (red) in the different study groups: WT (**A**–**C**), HMO-KO (**D**–**F**), TAU (**G**–**I**), HMO-KO+TAU (**J**–**L**); 40× magnification. Arrows point to astrocytes; hollow arrowheads point to macrophages. Number of retinas used in the experiment: WT *n* = 6, HMO-KO *n* = 6, TAU *n* = 6, and HMO-KO+TAU *n* = 6.

In the **WT group**, GFAP+ astrocytes showed a slight C3+ labelling, more evident in the somas and primary processes (Figure 11A–C). This labelling was not homogeneous throughout the retina, with areas of increased labelling and areas where no labelled cells were present. Macrophages were also stained with anti-C3 (Figure 11A,C).

**Figure 12:**
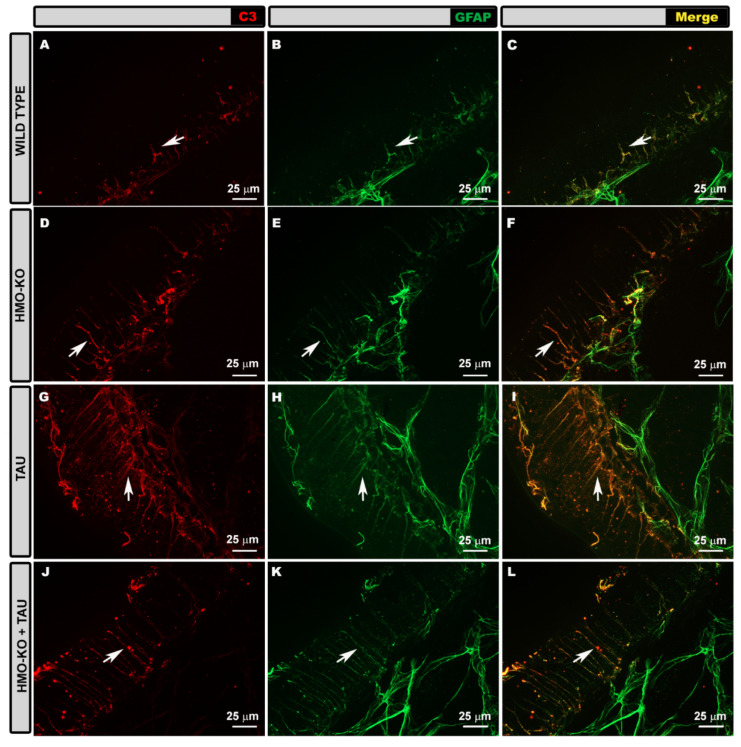
Retinal wholemount. Double immunohistochemical staining with anti-GFAP (green) and anti-C3 (red) in Müller cells in the different study groups: WT (**A**–**C**), HMO-KO (**D**–**F**), TAU (**G**–**I**), HMO-KO+TAU (**J**–**L**); 40× magnification. Arrows point to Müller cells. The image shows an edge of the retina, and therefore the entire thickness of the retina can be observed, allowing differentiation of the whole Müller cell as if it were a histological section. Number of retinas used in the experiment: WT *n* = 6, HMO-KO *n* = 6, TAU *n* = 6, and HMO-KO+TAU *n* = 6.

Müller glia can be seen in their entirety in the retinal wholemount when analyzing the areas where we made a cut in the retina to facilitate flattening. In these areas, the flattening of the tissue due to the pressure of the coverslip produces an image similar to that of a histological slice, allowing the cell to be analyzed in its entirety as it is arranged radially in the retina. The Müller cells that were observed, due to low GFAP staining, were lightly labelled with anti-C3 (Figure 12A–C).

In the **HMO-KO group** the C3+ labelling of GFAP+ astrocytes was similar to that observed in the WT group (Figure 11D–F). In Müller cells, C3 labelling was also like the WT group (Figure 12D–F).

In the **TAU group**, GFAP+ astrocytes showed more intense C3+ labelling, both in the somas and in the primary and secondary processes, than in the other study groups (WT, HMO-KO, HMO-KO+TAU) (Figure 11G–I). Although the marking was not homogeneous throughout the retinal extent, the extent of the marking was greater than in the WT group. The C3+ labelling of the Müller cells was also more intense than in the other groups, and the entire cell was distinguishable (Figure 12G–I).

In the **HMO-KO+TAU group**, the C3+ labelling of astrocytes was lower than that observed in the TAU group (Figure 11J–L). Müller cells had lower labelling than in the TAU group (Figure 12J–L).

#### 3.3.3. Quantitative Study of Retinal Macroglial Cells

##### Area of Retina Occupied by GFAP (AROA)

**Figure 13:**
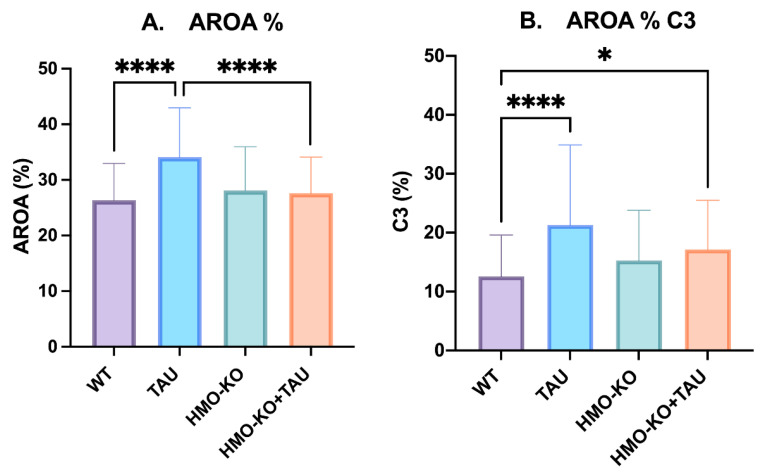
(**A**) Area of retina occupied by GFAP (AROA) in the different study groups. (**B**) Area of retina occupied by C3 (AROC3) in the different study groups. Data are expressed as mean values (±SD). One-way ANOVA; * *p* < 0.05, **** *p* < 0.0001. Number of retinas used in the experiment: WT *n* = 6, HMO-KO *n* = 6, TAU *n* = 6, and HMO-KO+TAU *n* = 6.

In the TAU group, there was a significant increase in AROA with respect to the WT group and to the HMO-KO+TAU group (*p* < 0.0001, in both cases) (Figure 13A).

##### Area of Retina Occupied by C3 (AROC3)

There was a significant increase in AROC3 in the **TAU group** with respect to the **WT group** (*p* < 0.0001) that was observed to a lesser degree in the **HMO-KO+TAU group** (*p* < 0.05) and was not observed in the **HMO-KO group**. In addition, a nonsignificant decrease in AROC3 was observed in the **HMO-KO+TAU** group with respect to the **TAU group** (Figure 13B).

## 4. Discussion

This study demonstrates for the first time that glial activation (macroglial and microglial) occurs in the retina in an aged mouse model induced with tauopathy in the hippocampus and how this glial activation was reduced in old mice KO for microglial HO-1.

In the present study, 15-month-old mice were used. This postnatal stage in mice is considered an early stage in the aging process [59]. In a previous study [24], WT mice of this age were found to have morphological signs of microglial activation such as a non-significant increase in the number of Iba-1+ cells in the OS layer, a non-significant decrease in the arborization area of Iba-1+ cells in the OPL, and a significant increase in the cell body area of Iba-1+ cells in the OPL, IPL, and NFL–GCL. In addition, numerous CD68+-labelled amoeboid-type Iba-1+ cells were found in the aged animals compared to no labelling in the young animals [24]. Likewise, in older (18–24 months) CX3CR1+/GFP transgenic mice, Damani et al. [60] found more retracted and less branched processes and a significant increase in microglia cell density in the OPL and IPL compared to young mice. These microglial changes observed with the aging are due to microglia being in a state of chronic activation, called *primed* microglia [26]. In the brain, these microglia increase the release of proinflammatory cytokines such as IL-1β, IL-6, or TNF-α, and they increase the expression of inflammatory receptors. They also increase their phagocytic capacity (CD68) and the expression of MHC-II [25,61]. Thus, microglia in aged mice undergo low-grade inflammatory changes and are in a chronically activated state compared to young adult mice [62]. This *primed* phenotype causes microglia to respond more acutely to damage, as has been demonstrated in aged mice induced with ocular hypertension (OHT) [24].

In our study, we used an aged animal model in which hippocampal tauopathy was induced by bilateral injection of AAV-hTau^P301L^. Tauopathies are associated with neurodegenerative processes in which an inflammatory process occurs with glial activation, cytokine release, synapse loss, axonal transport impairment, mitochondrial dysfunction, oxidative stress, DNA damage, and epigenetic changes, which promote cell apoptosis [63,64]. In the brain, there is microglial activation related to tauopathy [65,66]. PS19 transgenic mice (tauopathy model) aged 4 or 8 months showed increased microglial activation in the hippocampus, characterized by thickened and branched processes. This activated microglia was located near the neurofibrillary tangles [65]. In AD mice [67], microglial activation has also been found, with a significant increase in Iba-1+ cells and colocalization with T22 tau oligomers observed in the cerebral cortex of these mice. In 24-month-old Htau mice, a model expressing all six human tau isoforms without expressing mouse tau, a significant increase in activated microglia relative to WT, and co-localization of these microglia with tau [67] has been reported.

Nilson et al. [67] found microglial activation and morphological changes in the retina of P301L transgenic mice (tauopathy model). The authors observed microglial activation throughout the retina near the tau oligomer deposits. They found microglial hyperactivation and proliferation in the OS layer and in the retinal pigment epithelium layer. Morphologically, microglia in the eyes of P310L mice showed smaller cell bodies and irregular and short processes.

In our model of tauopathy induced by injection of AAV-hTau^P301L^ into the hippocampus, we did not find tau protein in the retina. Therefore, retinal microglial activation could come from brain microglial activation, since the retina is an extension of the brain [37]. Although there are studies that have detected the presence of tau aggregates at the retinal level in NDs such as AD [42,44], there are also studies that have not detected the presence of pathological aggregates of tau protein in the human retina of patients with AD or PD [68,69]. The absence of tauopathy in the retina in our model could be due to the fact that retinas were analyzed 35 days post-AAV-hTau^P301L^ injection. Therefore, this time line may not be sufficient for tauopathy to propagate to the retina

Our results of the morphometric study of retinal microglia showed a significant increase in the number of Iba-1+ cells and activated Iba-1 microglia in the OS layer, with no significant increase in other retinal layers. This increase of Iba-1+ cells in animals with tauopathy could be comparable to those found in retina and brain by other authors [67]. In addition, we found a significant decrease in the arbor area of Iba-1+ cells in the OPL and ILC in the TAU group, with more branched but thickened and retracted processes compared to the WT group. Process thickening and increased branching of processes was described by Yoshiyama et al. in the brain in PS19 transgenic mice [65]. In addition, retraction of microglial processes was also found in P301L transgenic mice [67]. We observed a significant increase in CD68 expression in microglial cells of the OS layer, OPL, and ILC in the TAU group with respect to the WT group. All this reveals that increased phagocytosis by microglia is occurring in the retina of animals with tauopathy, which would indicate an increased microglial inflammatory process [70].

In this study, we also found an increase of CD68+ dystrophic microglia in the OS layer in the TAU group with respect to the WT group. When the cell loses its capacity to maintain iron homeostasis, it becomes dystrophic microglia [71]. Dystrophic microglia have a lower neuroprotective capacity and can secrete a greater amount of proinflammatory molecules, being related to ND and neurodegeneration [16].

Macroglial changes (astrocytes and Müller cells) have also been observed in association with tauopathy. In the brain of 6-month-old PS19 mice (tauopathy model) [65], a significant increase in GFAP labelling has been found in the hippocampus, amygdala, entorhinal cortex, and spinal cord. Studies by Nilson et al. [67] showed in frontal cortical sections of mice with frontotemporal lobar dementia (FTLD) and AD a significant increase in GFAP labelling with respect to their control group. In addition, by ELISA technique, the authors found a significantly higher level of GFAP in FTLD and AD mice. In 11-month-old Htau mice [67], fragmented astrocytes with reactive morphology were found in the brain. In addition, in 24-month-old Htau mice [67], astrocyte activation was further increased when higher levels of tau oligomers were present.

In the retina of P301L transgenic mice with tauopathy, the presence of reactive astrocytes [67] located in proximity to tau oligomers has been detected. This has also been observed in the retina of 3xTg-AD (AD model) mice [72,73]. The retina of AD patients [74] showed astrogliosis at the level of the RGC layer, together with a significant increase in the area occupied by GFAP with respect to control patients. In addition, astrocytes significantly overexpressed C3 with respect to the control. All this was associated with increased levels of hyperphosphorylated tau aggregates.

In our study, as in the previous models, a significant increase in the area occupied by GFAP (AROA) was found in the TAU group with respect to the WT group, indicating the activation and hypertrophy of astrocytes [75,76]. A significant increase in the area occupied by C3 (AROC3) was also found with respect to the WT group, indicating that astrocytes were in a neurotoxic or A1 phenotype in which the secretion of proinflammatory cytokines, such as IL-1α or TNF, and complement factors, such as C1q or C3 [35], is increased. In Müller cells, more intense GFAP and C3 labelling was also detected in the TAU group than in the WT group. In 3xTg-AD mice (animal model of AD) Rodrigues-Neves et al. [72] found by Western blotting a non-significant increase in retinal GFAP levels at 4 months of age and a significant decrease at 8 months of age with respect to their WT groups. However, our study showed a significant increase in GFAP levels in the TAU group with respect to the WT group. This difference between the data may be due to the different animal models studied and the difference between the ages, as our mice were old (15 months). Regarding Müller cells, the authors [72] did not detect evident changes in the distribution and morphology of these cells.

In ND, the inflammatory process and, therefore, glial activation result in a worsening of the neurodegenerative process. Therefore, strategies are being developed to decrease this neuroinflammation to increase neuronal survival.

With aging and in ND, overexpression of HO-1 has been reported [57,77], as this enzyme is involved in the catabolism of the heme group, and one of its products is iron [47]; iron can accumulate and cause neuroinflammation.

In a study by Fernández-Mendívil et al. [55], it was found that in the brains of aged WT mice there was a higher expression of HO-1 than in adult WT mice, and this increase was even greater when exposed to inflammatory stimuli such as LPS. In these brains there was an increase in iron deposition, a primed microglia phenotype, and an increase in inflammatory markers such as iNOS, p65, IL-1β, TNF-α, Caspase-1, and NLRP3. However, in KO mice of HO-1 in microglial cells, all these alterations were prevented. In line with this study [55], we found that microglial deletion of HO-1 was protective for the retina of aged subjects, as indicated by a reduction of inflammatory markers.

In our results, we found that the number of microglial cells in the HMO-KO+TAU group was significantly smaller than in the TAU group in the OPL layer and non-significantly in the OS and ILC, which would indicate lower microglial activation and less tissue damage. The area of microglial cell arborization in the HMO-KO+TAU group was significantly larger than in the TAU group, indicating less process retraction and thus lower microglial activation in the HMO-KO+TAU group.

One fact that we do not know how to explain was the significant increase in microglial soma size in the HMO-KO and HMO-KO+TAU groups in the OPL; we do not know why HO deletion increased microglial soma size without influencing other signs of microglial activation.

The HMO-KO+TAU group had significantly fewer microglia with phagocytic capacity (CD68+) than the TAU group, indicating that less phagocytosis and thus less inflammation occurred in the HMO-KO+TAU group than in the TAU group.

All the above data demonstrated that microglial activation in animals with tauopathy but without HO-1 (HMO-KO+TAU) was much lower than in animals with tauopathy (TAU group).

We also demonstrated lower macroglial activation in animals with tauopathy but without HO-1 (HMO-KO+TAU group). Morphologically, the thickened and more branched appearance of astrocytes in the TAU group was less in the HMO-KO+TAU group, resembling more the WT group. AROA was significantly lower in the HMO-KO+TAU group with respect to the TAU group, being almost at the same level as the WT group. This indicates lower macroglial activation in the HMO-KO+TAU group with respect to the TAU group. We also found that AROC3 in the HMO-KO+TAU group was lower than in the TAU group (not significant), which would indicate that astrocytes might be in a somewhat less neurotoxic phenotype in the HMO-KO+TAU group than in the TAU group.

## 5. Conclusions

This study demonstrates that in the retinal tissue of animals with tau protein accumulations in the brain, there is a significant increase in signs of microglial and macroglial activation. However, these signs of activation are much lower in animals with tauopathy but without HO-1 in microglial cells.

These data have not been previously described and could open a window for the control of inflammation in tauopathy processes, as well as a target for drug development.

## Figures and Tables

**Table 1:** Antibodies employed for the immunostaining analysis and their corresponding information.

	Antibody	Concentration	Host	Reference	Company
**Primary antibodies**	Iba-1	1:600	Rabbit	01919741	Wako
	AT8	1:200	Mouse	MN1020	Thermo Fisher
	CD68	1:40	Rat	MCA1957GA	Bio Rad
	TAUY9	1:400	Rabbit	BML-TA3119-0100	Enzo lifesciences
	GFAP-GA5	1:150	Mouse	MAB3402	Millipore
	C3	1:15	Rat	HM1045	Hycult Biotech
**Secondary antibodies**	Alexa Fluor^®^ 405 Anti-Mouse	1:150	Goat	31553	Invitrogen
	Alexa Fluor^®^ 405 Anti-Rabbit	1:100	Goat	A31556	Invitrogen
	Alexa Fluor^®^ 488 Anti-Rat	1:150	Donkey	A21208	Invitrogen
	Alexa Fluor^®^ 488 Anti-Mouse	1:200	Goat	A11001	Invitrogen
	Alexa Fluor^®^ 594 Anti-Rabbit	1:800	Donkey	A21207	Invitrogen
	Alexa Fluor^®^ 594 Anti-Rat	1:500	Goat	405422	Biolegend

Abbreviations: ionized calcium binding adaptor molecule 1 polyclonal antibody (Iba-1); phospho-tau (Ser202, Thr205) monoclonal antibody (AT8); heavily glycosylated type I transmembrane glycoprotein antibody (CD68); Tau (human) polyclonal antibody (ATY9); glial fibrillary acidic protein monoclonal antibody (GFAP-GA5); complement component C3 monoclonal antibody (C3).

## Data Availability

The data supporting the findings of this study are available from the corresponding author upon request.
